# Improving model performance in mapping black-soil resource with machine learning methods and multispectral features

**DOI:** 10.1038/s41598-024-82399-3

**Published:** 2025-01-07

**Authors:** Jianfang Hu, Yulei Tang, Jiapan Yan, Jiahong Zhang, Yuxin Zhao, Zhansheng Chen

**Affiliations:** 1 Center for Geophysical Survey, China Geological Survey, Langfang, 065000 China; 2 Technology Innovation Center for Earth Near Surface Detection, China Geological Survey, Langfang, 065000 China; 3 China Aero Geophysical Survey and Remote Sensing Center for Natural Resources, Beijing, 100083 China; 4 College of Resources, Environment and Tourism, Capital Normal University, Beijing, 100048 China

**Keywords:** Black-soil resource, Soil mapping, Model performance, Machine learning, Ecology, Environmental sciences

## Abstract

Accurate information on the distribution of regional black-soil resource is one of the important elements for the sustainable management of soils. And its results can provide decision makers with robust data that can be translated into better decision making. This study utilized all Sentinel-2 images covering the study area from April to July in 2022. After masking clouds, all images were synthesized monthly. Based on the revised random forest classification algorithm, model performance using different feature combination programs were evaluated to search for an efficient, high-precision method for mapping black-soil resource. The impact on model performance of adding data from temperature, precipitation and slope geographic covariates was analyzed. And the robustness of the model was verified using Landsat-8 data with lower spatial resolution. The results showed that (1) the model based on multi-temporal ensemble features for mapping black-soil resource shows the best performance, with an OA of 94.6%; (2) adding temperature covariate can effectively improve the accuracy of black-soil resource mapping; (3) compared to the sentinel data, the performance of the model based on Landsat-8 data is reduced but still plausible, verifying the robustness of the model. This study provides a robust method to improve model performance for rapid mapping of black-soil resource.

**Supplementary Information:**

The online version contains supplementary material available at 10.1038/s41598-024-82399-3.

## Introduction

Black soil refers to suitable agricultural soil which has a black surface horizon, enriched with organic carbon that is at least 25 cm deep^1,2^. Black soil is recognized as one of the most fertile soils in the world, including three main soil groups Chernozem, Kastanozem and Phaeozem^3^. There are only four major black soil distribution areas in the world, which are in the Mississippi Plain of North America, the Pampas Plain of South America, the Northeast Plain of China, and the Ukrainian Plain^2,4^. Although black soils account for only one-sixth of the global arable land area, a significant percentage of oilseed, cereals, and tuber crops are harvested from black soils^5^. Apart from being highly productive lands, black soils are responsible for multiple ecosystem services. One of the most valuable services is the high potential to mitigate climate change because of their high soil organic carbon sequestration potential^6^. Due to anthropogenic intervention and intensive cultivation systems, black soils are suffering severe degradation such as erosion, loss of organic matter, compaction and soil acidification^7,8^. Considering their relevance for food security and their increasing vulnerability to soil degradation, it is of the utmost importance to promote the sustainable use, management and conservation of black-soil resource. Therefore, it is necessary to obtain accurate, updated, and detailed information regarding the distribution of black-soil resource.

Satellite remote sensing data has emerged as a vital tool for soil resource surveys and information generation. It contributes to the evolution of optimal land-use plans at scale ranging from regional to micro levels for sustainable development^9^. Traditionally, survey of black-soil resource based on ground surveys requires soil sampling at a large scale, which is relatively difficult and costly to implement^10,11^. Moreover, it is difficult to ensure the accuracy of the results in areas with large spatial heterogeneity. Satellite remote sensing data with rapid, low cost, and wide spatiotemporal coverage play a vital role in soil mapping by reducing the need for extensive time-consuming and costly field surveys^12–14^. Sahadevan explored the potential of multi-label classification approach to classify soil types using airborne hyperspectral image and Sentinel-2 images^14^. With the advancement of technology, multispectral images have the ability to obtain adequate soil information independently^15^. An ensemble modelling approach to improve the accuracy of soil texture component estimation using Sentinel-2 data has been investigated^16^. Multiple spectral transformation and topographic factors were also used by Neyestani to map soil class in central Iran, and their model had an overall accuracy of 53% and a kappa coefficient of 0.39^17^. However, the relationship between black soil reflectance spectroscopy and image information still needs to be further explored.

The properties of the soils that govern the spectral reflectance are color, organic matter, moisture, texture and mineralogy^9,18,19^. Soil organic matter content (SOMC) primarily controls the expression of black soil properties. Previous results have indicated that increasing SOMC results in an overall decrease in reflectance at the visible (VIS), near-infrared (NIR), and shortwave infrared (SWIR) spectral regions^20–22^. And most commonly used optical satellites, Sentinel-2 and Landsat-8, cover the relevant sensitive bands. Based on the relationship between soil properties and the spectral information of satellite images, many studies have accurately mapped the regional distribution of soil information^23^. For example, Lin explored a new way to accurately determine the SOMC by focusing on the influence of soil moisture on the SOM^24^. Luo used multitemporal synthetic Landsat-8 images in Google Earth Engine to obtain the regional mapping of SOMC^25^. A local strategy was proposed in the study of Meng to reduce the influence of the high heterogeneity of SOMC content and environmental variables on the prediction results^26^. And it was used to generate the first high-resolution global black soil region SOMC content product. Unfortunately, the band parameters extracted from ground spectra tend to suffer from the influence environmental noise. Spectral index based on linear or nonlinear band combinations can somewhat suppress the effects of factors such as soil surface conditions and the atmosphere^27,28^.

There is a need to further explore the potential of spectral index in efficient modeling of precise information on the extent distribution of black-soil resource. Time series images have been proven to be more effective than mono-temporal image for crop mapping, wetland mapping and land use mapping^29,30^. Previous studies extracted spatial and spectral parameters from time series remote sensing images to reflect more complete soil surface information, which is conducive to the development of soil classification mapping^31^. Consequently, establishing a relationship between time series spectral index and the soil properties enables the discriminating of spatial variations in black-soil resource^32^. To be based on large-scale, multitemporal, and high-dimensional input features, an effective model is essential. Random forest (RF), as a concrete implementation of the ensemble machine learning method, can effectively solve the problem of processing and analyzing remote sensing images with huge data volume and complex features^33–35^. Both the probability map and the categorical map of the black soil distribution were generated with the continuous improvements in computing platforms by using the RF model^36–38^. However, RF model is better at handling sensitive features, redundant features will reduce the model performance^38^. It’s necessary to develop an innovative framework combining a method for filtering key features that can best reflect the properties of the black-soil resource with the RF model.

The primary aim of this paper is to provide a more effective mapping model for black-soil resource, we focused on enhancing model performance through the utilization of readily available multispectral features and machine learning methods. To achieve this, the main objectives of the study are as follows: (1) study the methods of extracting and combining features from multitemporal remote sensing images for black soil mapping; (2) improve RF model by integrating feature filtering technology to increase the accuracy of black-soil resource discrimination. (3) Evaluate the reliability of adding slope data, temperature data and precipitation data for the mapping of black-soil resource. (4) Demonstrate the robustness of this model by applying it to Landsat-8 data. This study can provide a model reference for discriminating of black-soil resource by using easily available multispectral data and similar spectral curves. The resulting moderate-spatial-resolution (10-m) black-soil resource map can be used as technical support for soil monitoring and protection.

## Materials and methods

### Study area

**Fig. 1:**
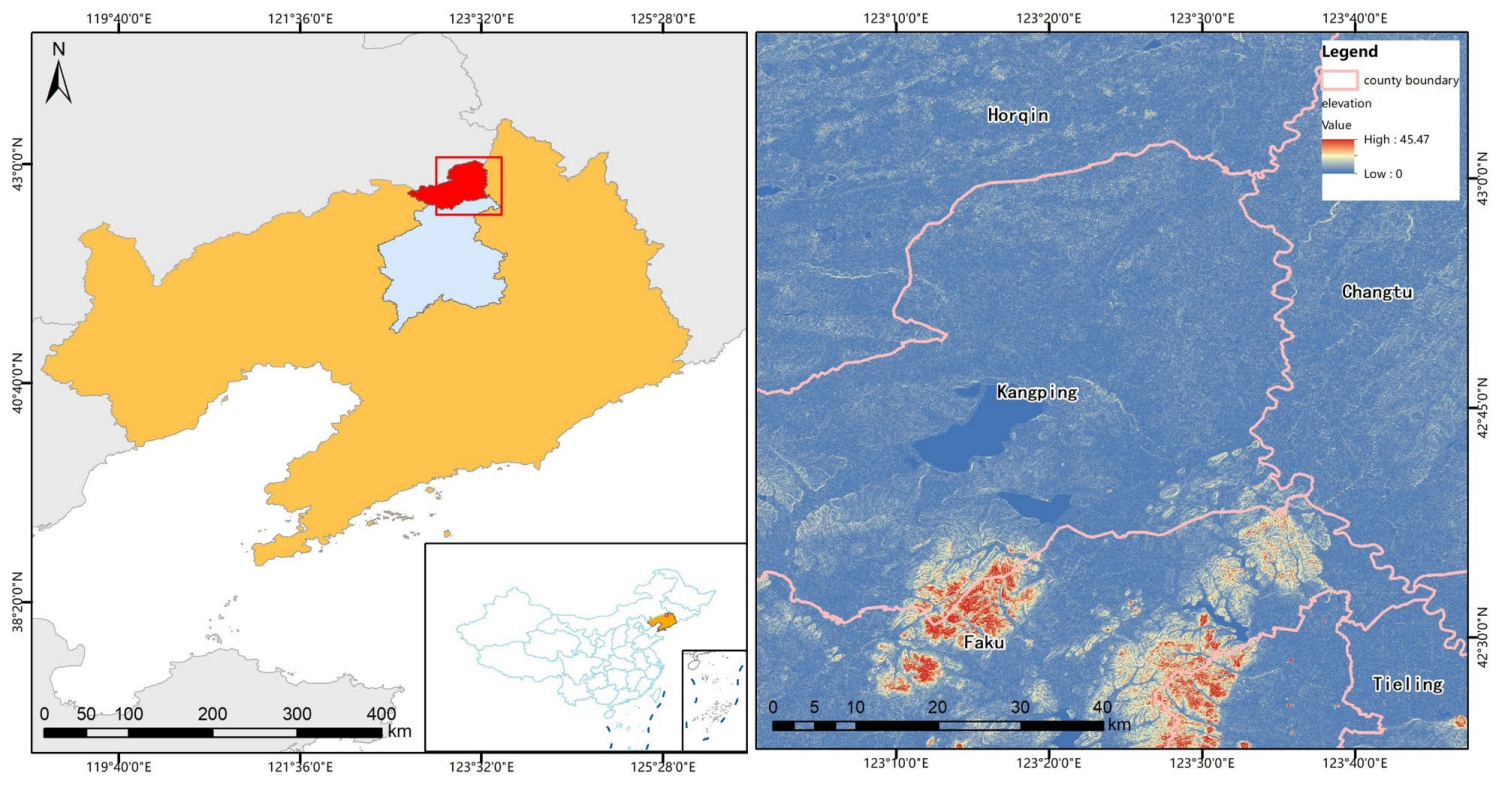
Overview of the study area Figures were created using QGIS (version 3.20, https://www.qgis.org/).

Figure 1 illustrates that the study area is mainly located in Kang ping County (131°27′–132°15′E, 46°28′–46°59′N) in the Liao River basin of Northeast China. The main towns covered by the study area are XCZ, HZWB, BSJZ and others (The information of towns is in Supplementary Table S1). The study area is located at the southern edge of the Horqin Sandy land, adjacent to Changtu and Faku counties, covering an area of over 4000 km^2^. The study area features a continental monsoon climate of the north temperate zone. The terrain of the study area is flat with an average elevation of 382.1 m^39^. The annual average temperature is 6.9 °C, the average sunshine time is 2867 h, the average wind speed is 4.6 m/s, the average precipitation is 524 mm, and the average frost-free period is 151 days^40^. The main types of black soil in study area are meadow soil, black soil, dark brown soil, etc. The area has a large plot area, suitability for large-scale planting. Sandy topography of the Horqin Sandy land poses a threat to black soil degradation in the study area^41^.

According to previous studies, the bare-soil period in study area lasts from the beginning of April to the end of May every year. From April to July, the region typically experiences a period characterized by low vegetation coverage, except for some areas covered by snow^30^. This facilitates the use of remote sensing satellites to monitor the physical and chemical properties of the black soil.

### Data acquisition and treatment

#### Training samples and verification samples

In recent years, our research group has conducted numerous field expeditions to the study area. The results of these field expeditions revealed a general view of the distribution and the characteristics of black soil resources in the study area. We classified the soil types in the study area as two dominant classes (black-soil and other-soil). Six different major land cover classes have been identified within the study area. The detailed description of each class was given in Table 1.

**Table 1:**
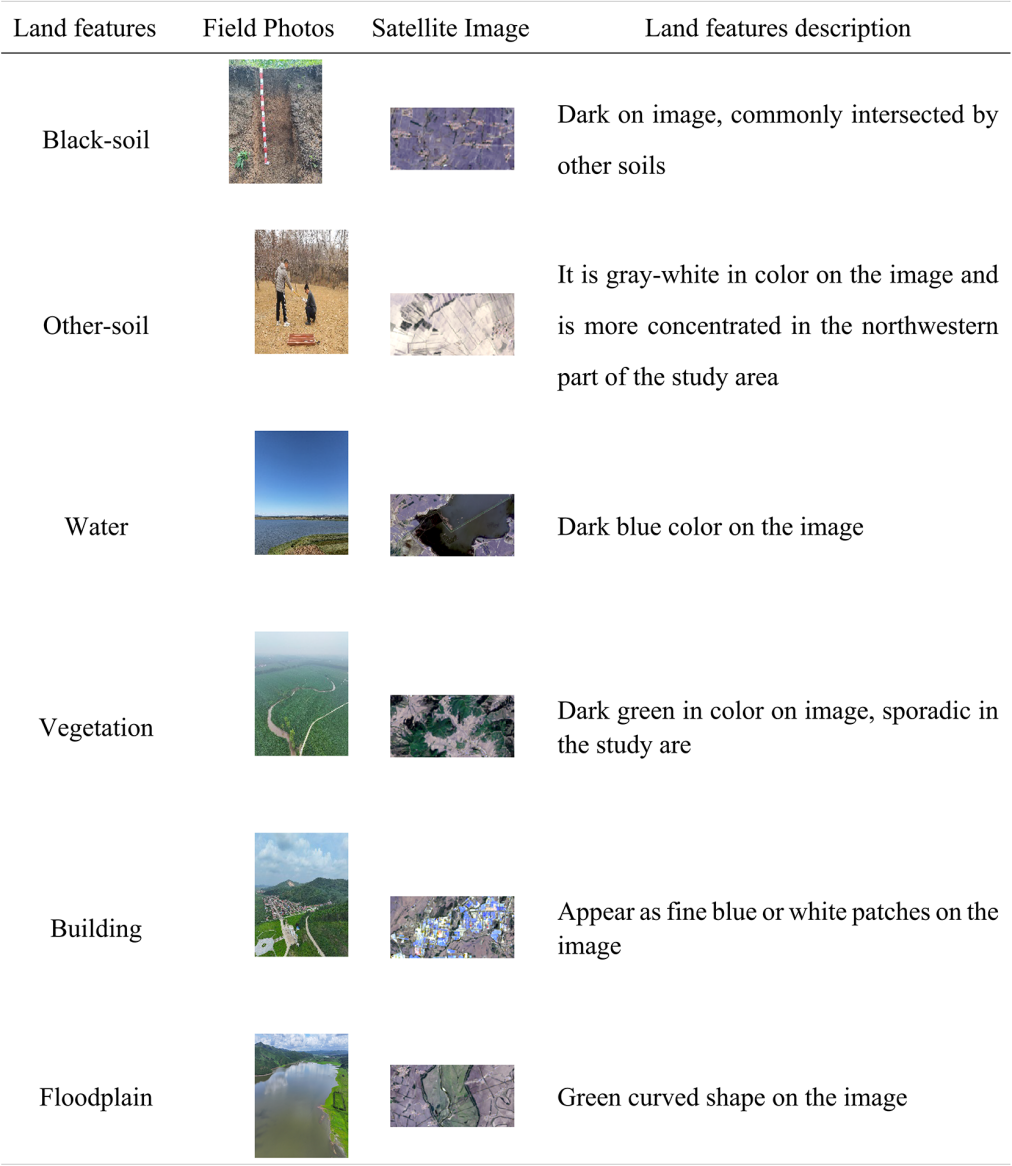
Detail description of land cover classes.

Both training samples and verification samples were collected from field sampling data in 2022. The sampling strategy used was stratified random sampling considering soil type, land cover types and other factors. Specifically, the study area was divided into six strata according to the land cover classes in Table 1. 10 m ×10 m pixels were designated as the smallest sampling unit, the number of sampling points within each stratum was determined based on the following ratio: “black soil: other-soil: water: vegetation: building: floodplain = 3:2:1:1:2:1”. The central position of the sample points was recorded with a global positioning system, and the soil color, soil thickness, SOC content of each sample were collected. The quality of training sample points has a strong relevance with the model performance. The sample points were filtered according to the soil thickness and SOC content, which is supplemented by Google Earth and high-resolution optical remote sensing images. And a total of 298 sample data were selected based on established classification systems for the study area (The information of sample data is in Supplementary Table S2). Among them, the total number of black soil sample points are 92.

#### Satellite retrievals

Sentinel-2 satellite data has the comprehensive advantages of high spatial and temporal resolution, rich spectral information and low acquisition cost. It provides a new opportunity for extracting information on the distribution of black soil on a large scale and with high precision. Sentinel-2 level-2 A (L2A) treatment level data product represents geometrically corrected and atmospherically processed surface reflectance data in 13 bands covering the visible to short-wave infrared with a spatial resolution of up to 10 m^42^ (The detailed information of the spatial resolution and spectral characteristics is in the Supplementary Table S3). According to the local climate and local crop phenology, the Sentinel-2 L2A imagery covering the study area from April to July in 2022 was selected to construct the experimental datasets (The image in June was excluded to avoid the impact on the model performance evaluation due to the high cloud content of the June image). The Sentinel images were acquired from GEE platform (https://developers.google.com/earth-engine/datasets/catalog/COPERNICUS_S2_SR_HARMONIZED).

**Table 2:** Image collection information.

Image data	Date	Cloud cover (%)	Image data	Date	Cloud cover (%)
Sentinel-2	13-Apr-22	4.95	Landsat-8	07-Apr-22	0.5
	18-Apr-22	0.79			
	18-May-22	0.9		09-May-22	7.1
	22-May-22	0.4		26-Jun-22	5.35
	22-Jul-22	2.92		19-Jul-22	4.96

In order to compare the performance of the black soil resource mapping model applied in different satellites, Landsat-8 data covering the study area from April to July 2022 were selected from GEE platform (https://developers.google.com/earth-engine/datasets/catalog/LANDSAT_LC08_C02_T1_L2). The spatial resolution of Landsat-8 data is 30 m, and the revisit period is 16 d (The detailed information of Landsat-8 data is in the Supplementary Table S4). Table 2 shows the information of acquisition date and cloud content for the Sentinel-2 data and Landsat-8 data used in this study.

All Sentinel-2 images were cloud masked using the ‘QA60’ band in the image to obtain a cloud-free time-series Sentinel-2 image collection. Among them, ‘Bit10’ and ‘Bit11’ were used to identify the opaque clouds and cirrus clouds. Then, the ‘QA_PIXEL’ and ‘QA_RADSAT’ bands were used to mask the images with clouds and shadows and obtain a cloud-free Landsat-8 image collection for the time period of interest. A median composite was performed based on each monthly image on the cloud-free Sentinel-2 image collection and cloud-free Landsat-8 image collection.

#### Geographic covariates

Many previous studies have used supplemental topographic and climate data to perform soil discriminating model, and the results have shown that the slope, average temperature and average precipitation are the most useful factors for improving the performance of soil discriminating model with good interpretability^43^. The accumulation of organic matter differs significantly between steep areas and flat terrain^44^. The air temperature affects the soil temperature, and soils with different temperatures will have different spectral characteristics^45^. Precipitation directly affects soil water content and further affects the soil reflectance spectral characteristics^46^.

Three geographic covariates were selected in this study, with data from the GEE platform. For the slope data, we used NASADEM Digital Elevation 30-m data to calculate “SLOPE” as the slope input of the study area. The average temperature and precipitation data were calculated from the “ERA5 Monthly Aggregates”. All geographic covariate data were resampled to ensure uniform spatial resolution (10–30 m) as input to the model.

### Selection of feature variables

Table 3 describes in detail the feature variables extracted in this study, which consist of two main parts: band-features and spectral index-features.

**Table 3:** Feature variable set (variables in ATSAVI mean that X = 0.08, a = 1.22, b = 0.03).

Feature set	Feature variables	Formula	References
Band-features	VIS, NIR	B2-9, B8A, B11; B2-7	Masek JG, Claverie M, Ju J, et al. (2015)^47^
Soil reflectance index	GOSAVI	$\:\frac{NIR-G}{NIR+G+0.16}$	Sripada RP, et al. (2005)^48^
	NDSI	$\:\frac{SWIR\:1-SWIR\:2}{SWIR\:1+SWIR\:2}$	G. A R, D. K H, V. V S. (1994)^49^
	ATSAVI	$\:a\frac{NIR-a\cdot\:RED-b}{a\cdot\:NIR+RED-a\cdot\:b+(1+{a}^{2})}$	Huete AR. (1988)^50^
Vegetation index	NDVI	$\:\frac{NIR-RED}{NIR+RED}$	Yao B, Gong X, Li Y, et al. (2024)^51^
	RVI	$\:\frac{NIR}{RED}$	Birth GS, Mcvey GR. (1968)^52^
	RDVI	$\:\frac{NIR-RED}{{(NIR+RED)}^{0.5}}$	Liang-Pengyin YI, (2004)^53^
Red-edge index	NDVIre1	$\:\frac{NIR-re1}{NIR+re1}$	Luciano S, Richard F, John S, et al. (2011)^54^
	NDVIre2	$\:\frac{NIR-re2}{NIR+re2}$	
	NDVIre3	$\:\frac{NIR-re3}{NIR+re3}$	
Other-index	NDMI	$\:\frac{NIR-SWIR}{NIR+SWIR}$	Bin LI, Huimin W, Mingzhou Q, et al. (2017)^55^
	NDBI	$\:\frac{SWIR-RED}{SWIR+RED}$	Chen XL, et al. (2006)^56^
	MNDWI	$\:\frac{G-SWIR}{G+SWIR}$	Sahoo, Shashikanta, Setia, et al. (2015)^57^

The color of black soil and the content of SOMC significantly affect the band reflectance. In this study, the reflectance of 10 bands of multi-temporal images was selected as the band-features. The adding of the water index not only enhances the spectral response of soil moisture and amplifies the subtle differences in the spectral properties of the black soil, but also suppresses the effect of and floodplains on soil reflectance^58^.Soil reflectance indexes are closely related to the type of soil and the SOC content, three typical soil reflectance indexes were included in the feature variable set. According to the land cover types in the study area, vegetation index and building index are extracted from the preprocessed Sentinel-2 data. In addition, the red-edge index was taken as a separate category of feature in this study.

### Algorithm descriptions

Random Forest (RF), first proposed in 2001, is a regression and classification decision tree approach widely used in digital soil mapping^59,60^. RF uses multiple trees to train and predict samples, and its essence is to use multiple decision-making classifiers to determine the final classification result. This model has obvious advantages in processing high-dimensional data compared with other decision tree classification algorithms. The RF classification model and the ‘ee. Classifier. smileRandomForest ()’ program were employed in GEE to map black-soil resource. As a result of repeated trials, the parameters are set as follows: the number of trees was set to 100, mainly to balance the accuracy and operation efficiency; for VariablesPerSplit, we used the default value of ‘null’; for minLeafPopulation, we used the default value of 1; for bagFraction, we used the default value of 0.5; for maxNodes, we used the default value of ‘null’; and for seed, we used the default value of 0.

Recursive Feature Elimination (RFE) method is a feature selection method commonly used in machine learning^61^. It works by recursively removing features and building models on the remaining features until the optimal subset of features is found. It initializes the required feature set to the entire data set, calculate the importance of the features^62^. Random Forest Feature Importance, i.e. Tree’s Feature Importance from Mean Decrease in Impurity (MDI) were calculated in this study. At each iteration, the least significant features are removed based on their importance scores determined by the model. In order to identify the most relevant features for mapping while reducing overfitting and improving model interpretability^63^, This study improved RF model by combining RFE method to develop a multi-feature ensemble random forest model. The feature elimination process continues until the specified number of features is reached or until the performance of the model no longer improves.

In order to explore the optimum black-soil resource mapping model, four models were designed based on the features in Table 3. The fundamental feature combination model (NBF-RF) was constructed using the mono-temporal band reflectance. In order to eliminate the influence of other factors, such as atmosphere, on the information of image bands, we added spectral index features to design the mono-temporal ensemble features model (NEF-RF). Multi-temporal ensemble features model (MEF-RF) was constructed to demonstrate the validity of time series features. In addition, multi-temporal band features model (MBF-RF) was constructed to compare with the MEF-RF model to demonstrate the necessity of multispectral features. Information on the models is shown in Table 4. We evaluated the accuracy of these models to investigate the effect of different feature variables and obtain the best model performance.

**Table 4:** Description of the models.

Model	Describtion
NBF-RF	Mono-temporal band reflectance
NEF-RF	Mono-temporal band reflectance and spectral index features
MBF-RF	Multi-temporal band reflectance
MEF-RF	Multi-temporal band reflectance and spectral index features

### Accuracy assessment

We evaluated the models via independent verification methods. First, we used filter algorithm to divide sample points into training samples and verification samples in the ratio of 7:3. Ultimately, 70% of the samples (209) were utilized to develop the mapping model, the remaining samples (30%) were utilized to assess the performance of the model. A confusion matrix was developed using the outputs and verification samples for this comparative analysis. The confusion matrix is a widely used method for accuracy evaluation in land cover classification. In confusion matrix, the values of verification samples are compared with the values of the corresponding result data to provide categories like a true positive (*TP*), true negative (*TN*), false positive (*FP*), and false negative (*FN*)^64,65^. Finally, parameters like *recall* and *F1* score can be computed to evaluate model performance (Eqs. (1)–(3)).

1 $$precision=\frac{{TP}}{{TP+FN}}=\frac{{TP}}{{Total~predicted~positive}}~$$

2 $$recall=\frac{{TP}}{{TP+FN}}=\frac{{TP}}{{Total~actrual~positive}}~$$

3 $$F1=2*\frac{{precision*recall}}{{precision+recall}}$$

4 $$OA=\frac{{TP+TN}}{T}$$

In order to visually and concisely compare the overall performance of different models (Table 4), The Overall Accuracy (OA) were employed in this study. Equation (4) states the OA computed for further statistical analysis, where *T* is the total number of pixels in the image. It is the ratio of the number of correctly classified image elements to the total number of image elements, representing the classification accuracy of the overall image elements. Several field expeditions were conducted for ground-truthing of different land use land cover types, as described in Table 1 (Section “Data acquisition and treatment”). The ground truth of different land cover types is presented in supplementary Fig. 2.

In response to the map we obtained, we conducted another field survey of the study area. We intend to verify the accuracy of the map through field research (The survey route is shown in the supplementary Fig. 3). Meanwhile, Reference distribution map for different classifications were further derived from all the field sampling data using random forest interpolation, which was used to design an overlay analysis of the reference map against the map obtained from the model. In addition, we conducted statistical analyses for black-soil source using a categorized statistical tool in QGIS.

## Results and discussion

### Spatial distribution of black-soil resource

Model with the highest accuracy among the algorithm described in the previous section was used to map the spatial distribution maps of the black-soil resource in the study area. The results for the overlap of the same classification show that the accuracy of the map obtained by the model achieved 90%. In addition, the areas in the reference and simulated maps obtained by the categorized statistical tool indicated a consistency of more than 90% in the black-soil areas. These comparisons support the fact that the map we acquired has reliable accuracy.

**Fig. 2:**
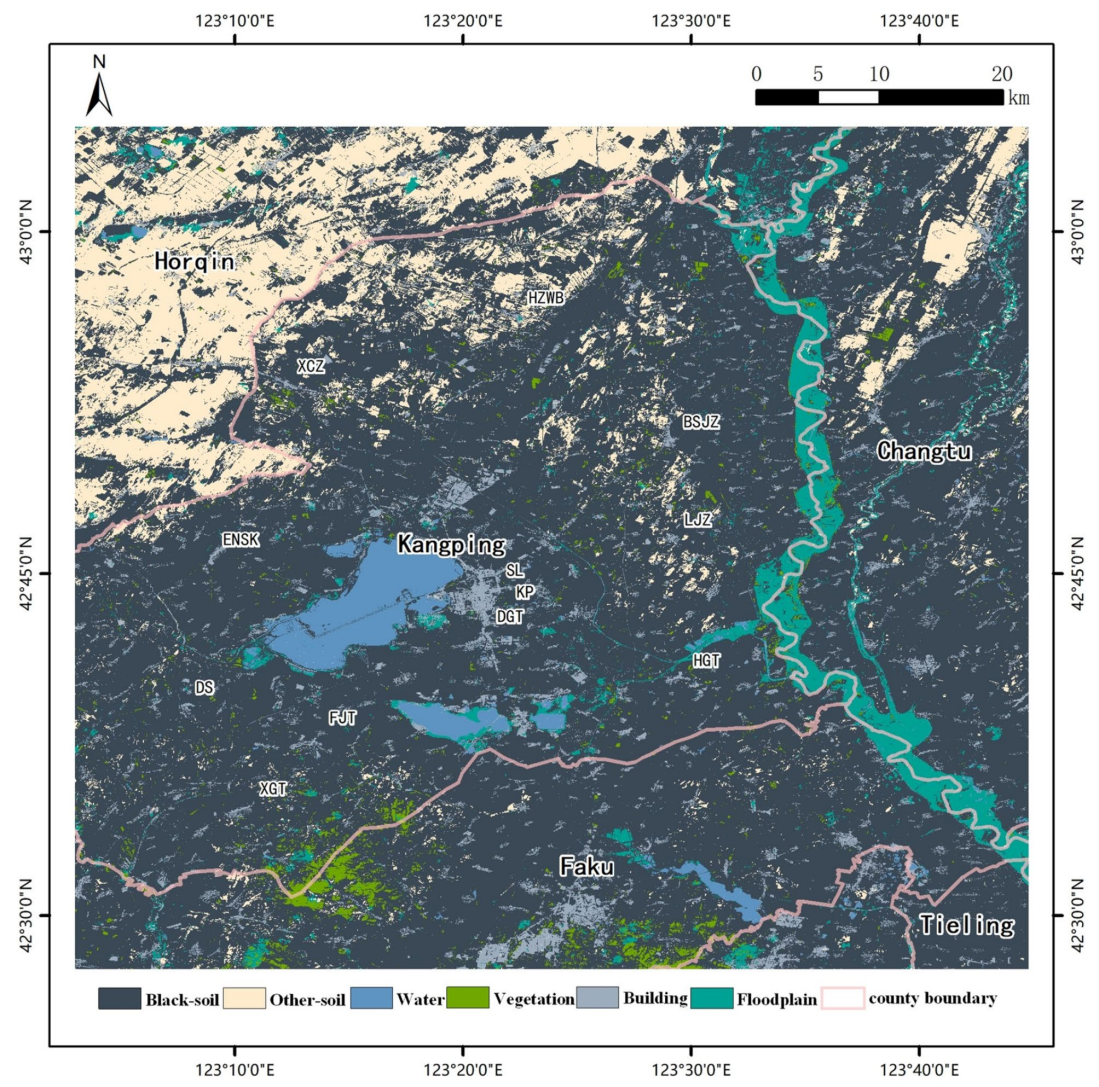
Spatial distribution of black-soil resource Figure was created using QGIS (version 3.20, https://www.qgis.org/).

Black-soil is primarily concentrated in the southern part of the study area, with a high concentration in DX, XGT, FJT, DGT, and ENSK in the southwestern region and KP, SL in the central region (Fig. 2). However, the distribution of black-soil in the northwest part of the study area, specifically in Horqin District, and in XCZ, HZWB, BSJZ, and LJZ at the northern part of Kangping town, appears to be fragmented. This may be due to the impact of soil erosion at the southern edge of the Horqin Sandy land (The towns mentioned can be found in Supplementary Table S1).

### Model performance evaluation

#### Model performance based on NBF-RF

**Fig. 3:**
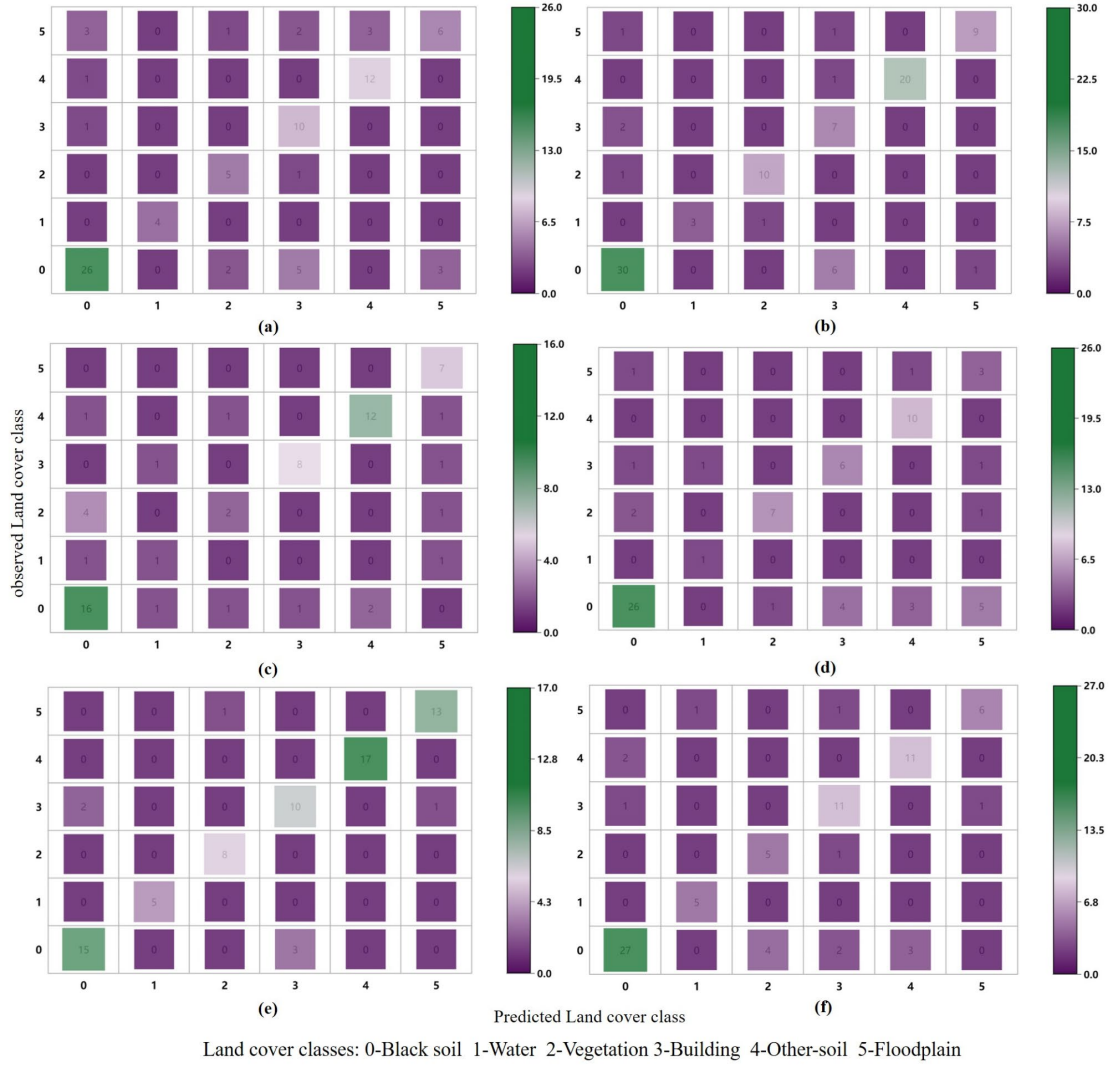
Confusion matrix of a model produced outputs using (**a**) NBF-RF model in April (**b**) NBF-RF model in May (**c**) NBF-RF model in July. (**d**) NEF-RF model in April (**e**) NEF-RF model in May (**f**) NEF-RF model in July.

The evaluation results of the model based on NBF-RF are shown in Table 5. The model constructed based on the image in May for discriminating black-soil resource had the highest accuracy, with an OA of 79.7%. The confusion matrix generated using the NBF-RF model (Fig. 3) showed that several land cover classes were misinterpreted. In particular, the model based on April had more misidentification of ‘black-soil’, and the model based on May showed significant improvement. However, there are still significant amounts of ‘black-soil’ were misinterpreted as ‘building’. Statistical analysis was carried out to evaluate the accuracy of the land cover map using the F-1 score indicators. The F-1 score of ‘black-soil’ based on the model in May presented the highest value. The OA of NBF-RF model in different months are still unstable. It is necessary to explore the effect of the inclusion of spectral indices on model accuracy and stability.

**Table 5:** Model performance based on mono-temporal features.

Land cover	NBF-RF			NEF-RF		
	April	May	July	April	May	July
	F-1 score					
Black-soil	0.66	0.84	0.74	0.68	0.85	0.81
Water	0.91	0.85	0.63	0.89	0.88	0.79
Vegetation	0.62	0.83	0.63	0.78	0.84	0.66
Building	0.89	0.79	0.84	0.76	0.76	0.78
Other-soil	0.81	0.97	0.82	0.87	0.86	0.81
Floodplain	0.63	0.85	0.77	0.65	0.92	0.79
OA (%)	75.0	79.7	75.3	77.9	81.1	75.8

**Fig. 4:**
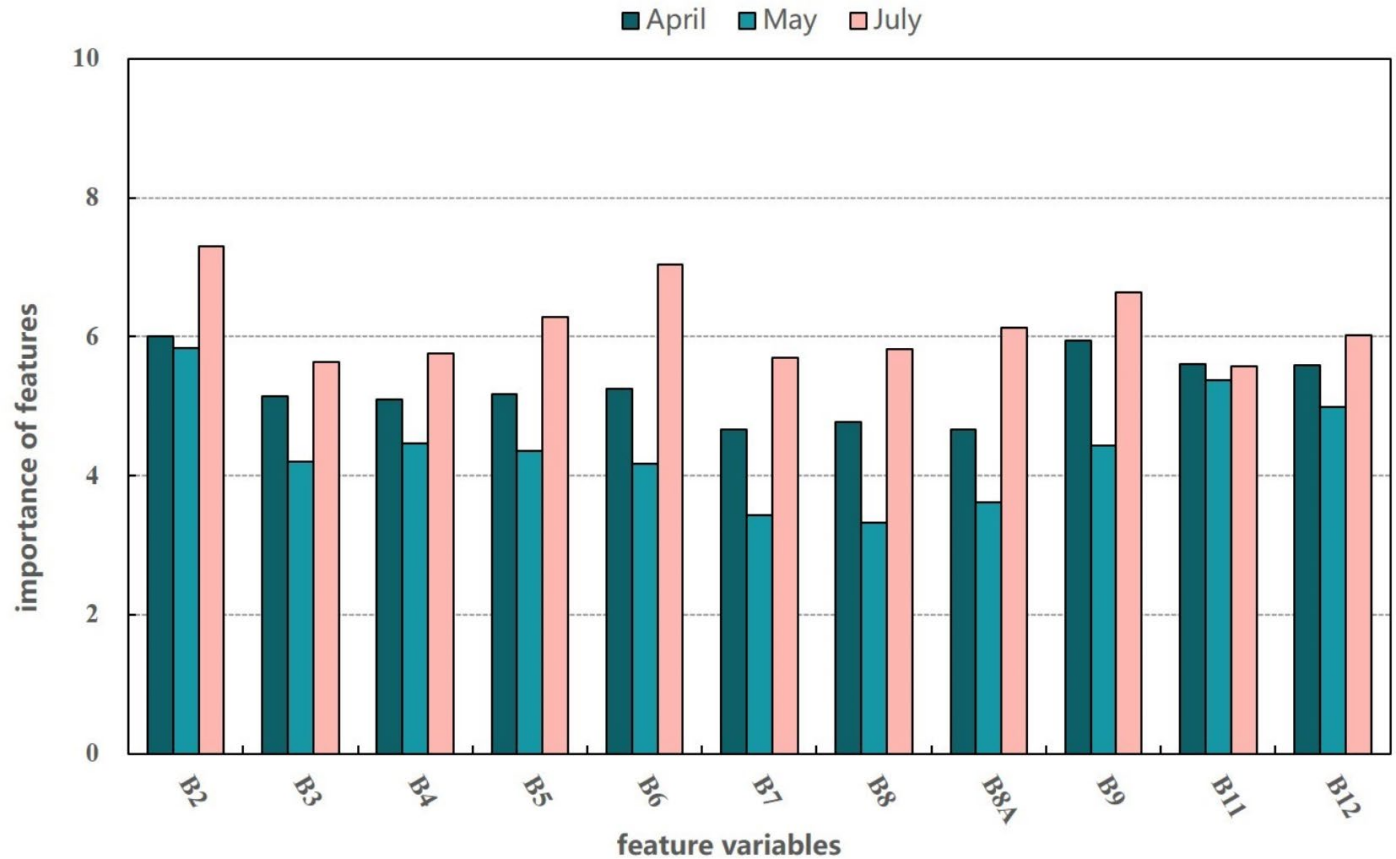
Importance of mono-temporal band features.

To better understand the impact of band features on accurately determining black-soil resources, we obtained the importance of band features from each individual image taken at a single point in time. As shown in Fig. 4, band features of B2, B9, B11, and B12 contributed more to the identification of black-soil resource. It is due to the fact that the spectral profile of black soil has significant absorption valleys in the red visible band and shortwave infrared band (350 ~ 700 nm, 1400 ~ 2052 nm), which exactly correspond to these bands. The low importance of B8A and B7 indicate that the vegetation red-edge bands do not have a significant effect on improving the discrimination of black-soil resource.

#### Model performance based on NEF-RF

The overall performance made by the NEF-RF model in each month improved the output result significantly compared to the performance made by NBF-RF model, with OA increased by 2.9, 1.4, and 0.5 respectively (Table 6). It indicates that the inclusion of spectral index can effectively suppress the influence of atmosphere and other factors to effectively improve the model performance. The NEF-RF model constructed in May performed equally well as the NBF-RF model in terms of accuracy. It indicated that May is the optimal time for discriminating black-soil resource. The problem of misinterpretation of land cover classes significantly improved when the NEF-RF model was applied. For instance, the “Black-soil” land feature is misinterpreted as a “Vegetation” or ‘Building ’land feature when using the NBF-RF. The NEF-RF model provided better differentiation between above land cover classes, which was misclassified in the NBF-RF model. The results of the statistical analysis carried out in this model are presented in Table 6. Compared to the NBF-RF model, the F-1 score of “Black soil” in the NEF-RF model increased but at a lesser amount.

**Fig. 5:**
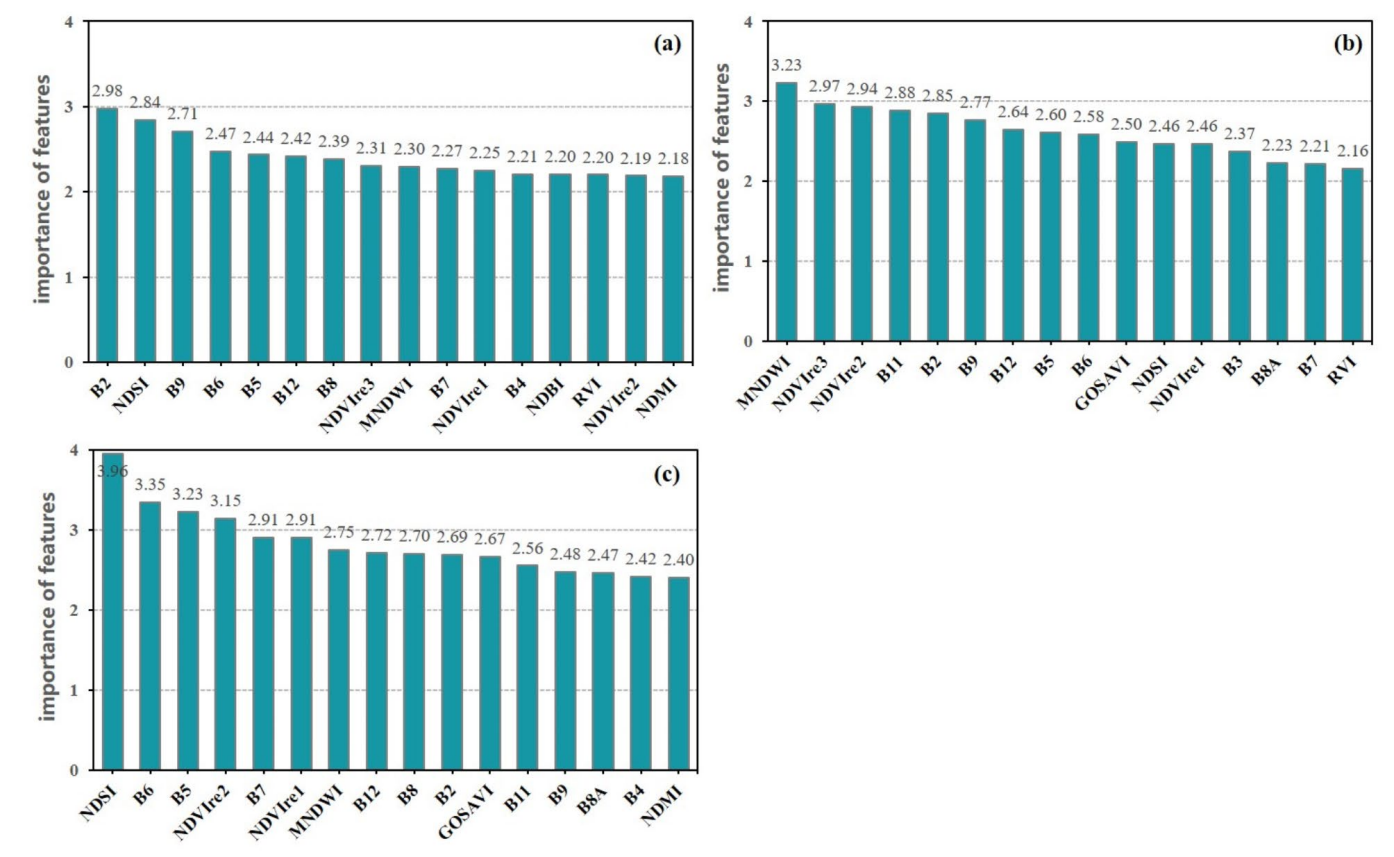
Importance of mono-temporal ensemble features (**a**) in April (**b**) in May (**c**) in July.

The importance order of the ensemble features in different periods is slightly different. But the importance of the band features (B2, B5, B6, B12) and the index features (MNDWI, NDSI, NDVI) extracted from different phase images are at a high level (For a clearer presentation, the 16 feature variables with higher importance in each month are shown in Fig. 5). From the view of the top ten features ranked in the combination of mono-temporal ensemble features, the ratio of band features has reached more than 60%. Band reflectance of satellite images can effectively reflect the characteristic difference information of black-soil resource, which is very important for the identification of black-soil resource.

#### Model performance based on multi-temporal features

The evaluation results of the MEF-RF model with or without feature filtering are shown in Table 6. Compared with the model performance using only band features or mono-temporal ensemble features, the performance of the MEF-RF model has been improved significantly. Before feature filtering, the OA of the black-soil resource discrimination model reached 91.4%. The data redundancy was reduced based on the RFE feature selection method. The iteration ended until the OA increased is less than 1%. It is considered that the model reached the most stable state when 33 features were selected (Supplementary Fig. 4). And the accuracy of the black-soil resource identification model was higher after feature filtering, with an OA of 94.6%. Extracting the same band features and index features based on images from different periods can provide richer image information to effectively solve the problem of different sensitivity of reflectance spectra affected by time.

**Table 6:** Model performance on multi-temporal features model.

Feature combination	Feature filtering	Number	OA (%)
Multi-temporal ensemble feature	None	126	91.4
	RFE	33	94.6
Multi-temporal band feature	None	34	85.7
	RFE	33	90.4

**Fig. 6:**
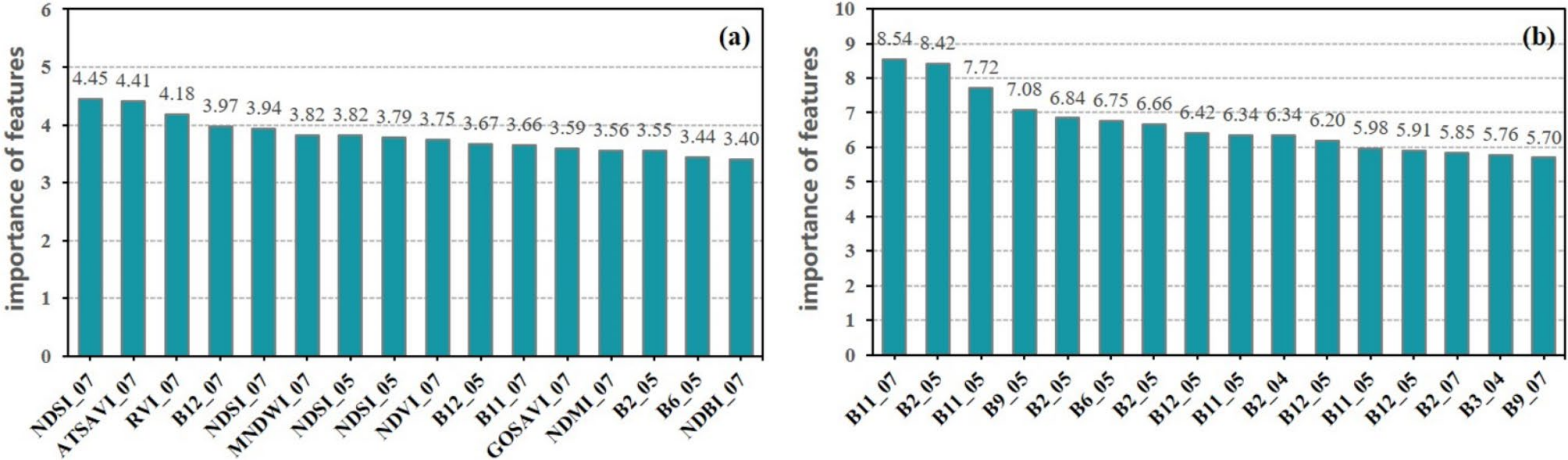
Importance of (**a**) multi-temporal ensemble features (**b**) multi -temporal band features.

Figure 6 illustrates that, among the various time-phase ensemble features with high importance order, the spectral index features, especially the soil reflectance index and vegetation index, carry more significance. Spectral indexes derived from the combination of reflectance values across multiple bands can effectively enhance the correlation between black soil characteristics and sensitive band features. The bands comprising the soil reflectance index exhibit heightened sensitivity to the properties of black soil, which makes the soil reflectance index a significant contributor to enhance the model performance in mapping black-soil resource.

**Fig. 7:**
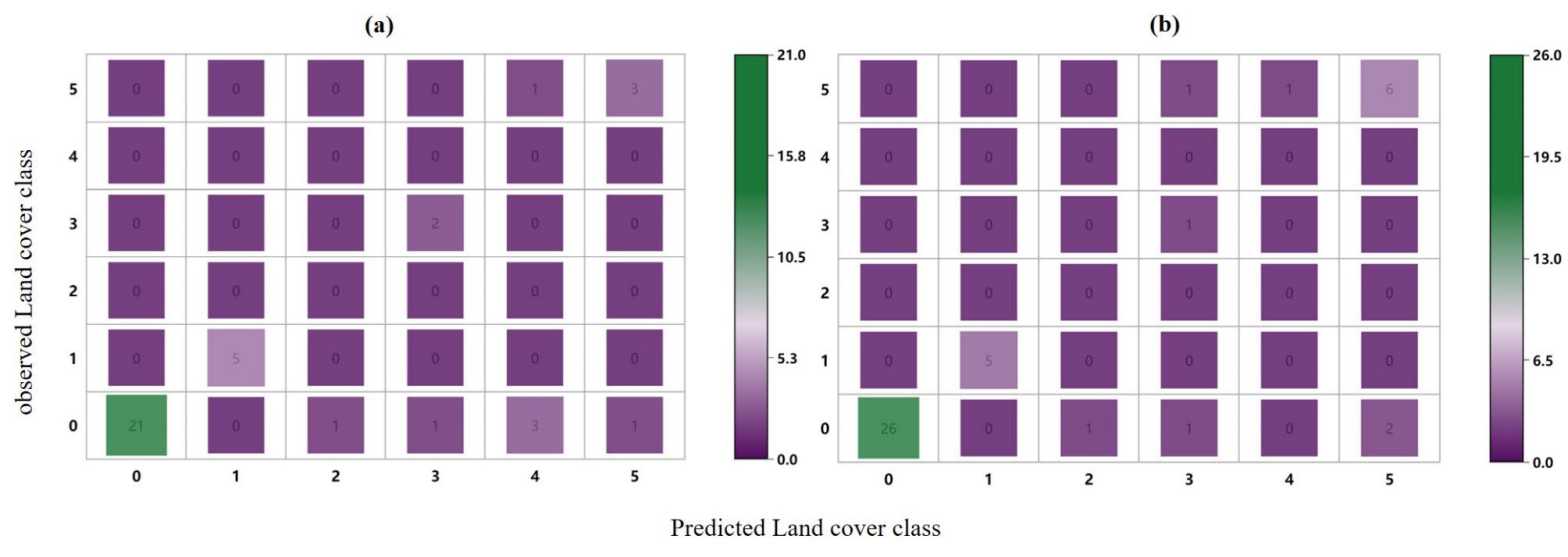
Confusion matrix of a model produced outputs using (**a**) NBF-RF model (**b**) NEF-RF model.

We further compared the performance of the MEF-RF model with that of the MBF-RF model. Obviously, the MEF-RF model performed better (Table 7). After feature filtering, 33 selected features were used in the MBF-RF model, achieving an OA of 90.4% (Supplementary Fig. 3 recorded the relationship between the MBF-RF model performance and the number of features). The confusion matrices generated using the multi-temporal feature models (Fig. 7) showed a significant reduction in misinterpreted, compared to the results based on the mono-temporal model. There was no significant difference in the confusion matrices of MEF-RF model and MBF-RF model. Specifically, there was a reduction in the misinterpretation of ‘black soil’ as ‘other soil’ in the MEF-RF model compared to the MBF-RF model. The F-1 score of the MEF-RF model were also increased compared to the MBF-RF model (Table 7).

In terms of the importance of multi-temporal band features, those from May had a high percentage. It’s due to the fact that spring plowing of cropland in the Northeast was mostly complete in May, cropland straw cover was low, and the high soil moisture period from snowmelt had ended. Although the importance of vegetation red-edge band ranked low in mono-temporal band features, its contribution in July was higher due to the high vegetation cover, improving the model performance (Fig. 6).

### Effects of geographic covariate data on model performance

We evaluated the model performance by adding geographic covariates based on the best black-soil resource discriminating model. The model performance varied with different geographic covariates, indicating their differing roles in black-soil discrimination. Including precipitation and slope covariates decreased model accuracy, indicating these variables do not significantly contribute to black-soil discrimination and introduce data redundancy. The addition of temperature covariate to the MEF-RF model resulted in a slight improvement in model performance, with OA increasing from 94.62 to 95.71% (Table 7). However, the F1 scores of ‘black soil’ in the model decreased slightly after adding the covariates. This illustrates that the model with geographic covariates indirectly improved the performance in mapping black-soil resources by improving the ability to identify other land cover classes.

**Table 7:** F-1 score on models.

Land cover	MEF	MBF	GC	Landsat
Black-soil	0.98	0.94	0.91	0.89
Water	0.91	0.89	1	0.89
Vegetation	0.85	0.76	0.88	0.92
Building	0.78	0.75	0.83	0.8
Other-soil	0.76	0.87	1	0.88
Floodplain	0.79	0.85	0.94	0.67
OA (%)	94.62	90.40	95.71	90.01

**Fig. 8:**
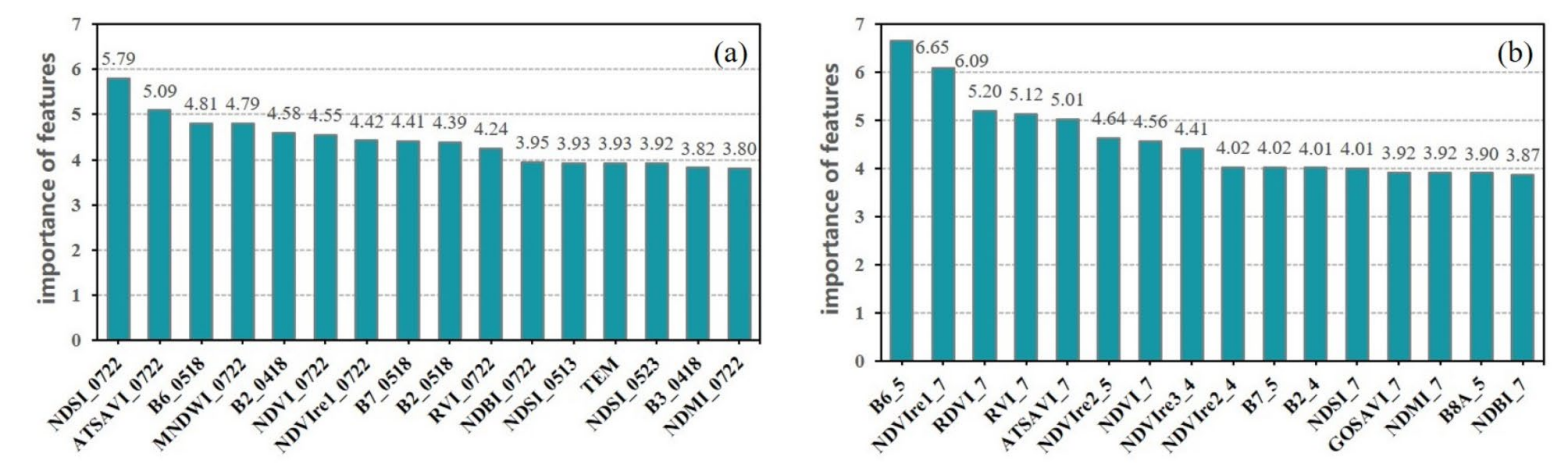
Importance of (**a**) MEF-RF model with geographic covariates (**b**) MEF-RF model using Landsat-8 data.

To further discuss the effect of geographic covariate data on model performance, we obtained the importance of band features after adding geographic covariates. The result in Fig. 8 shows that temperature covariate is of high importance in black-soil discriminations.

### Model robustness evaluation

**Fig. 9:**
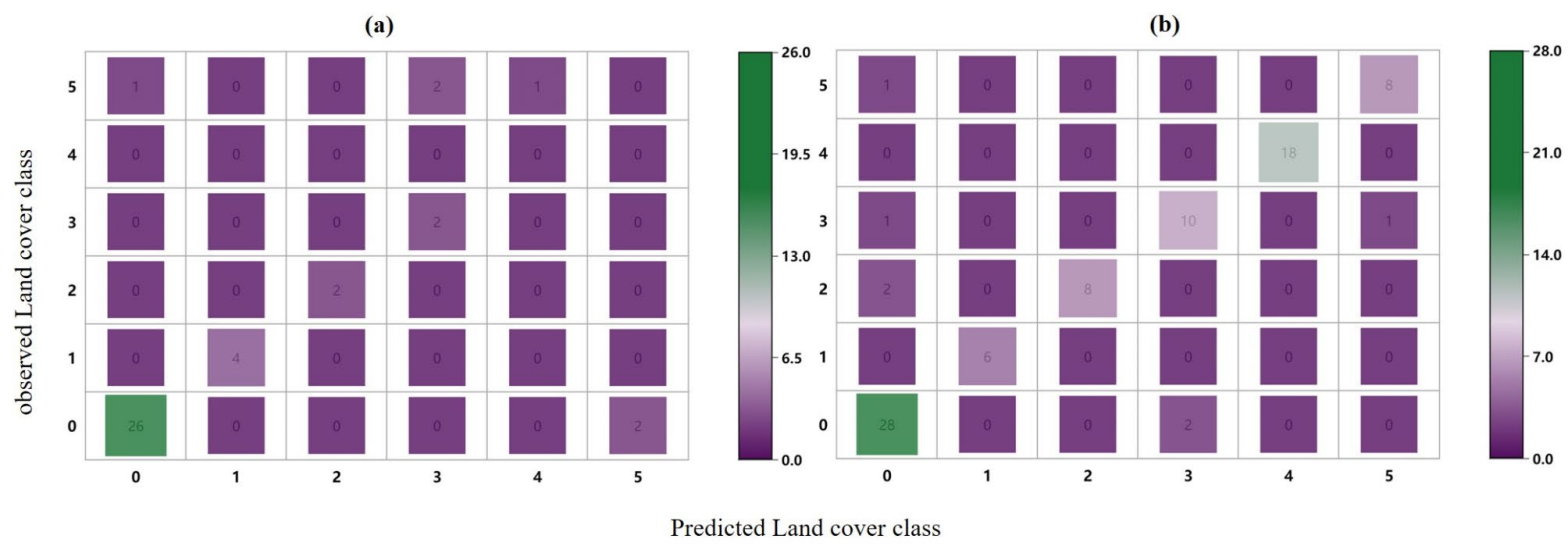
Confusion matrix of a model produced outputs using (**a**) MEF-RF model adding geographic covariates (**b**) MEF-RF model with Landsat-8 data.

The model performance with Landsat-8 data applied was evaluated to validate the robustness and transferability of the MEF-RF model for discriminating black-soil resource. In comparing the accuracy of the models constructed based on Landsat-8 data and Sentinel data, we observed the accuracy of the MEF-RF model based on Landsat-8 data is slightly lower than the MEF-RF model based on Sentinel-2 data, with an OA of 90.01% (Table 7). This is mainly due to the gap in spatial resolution of different remote sensing data. However, the MEF-RF model based on Landsat-8 data performed better than the other models in this study. The confusion matrix generated using the MEF-RF model with Landsat-8 data also showed a low misclassification rate (Fig. 9). It was demonstrated that the model developed in this study still performs well with limited spatial resolution. This suggests that the wider availability of the MEF-RF model for black soil discrimination.

**Fig. 10:**
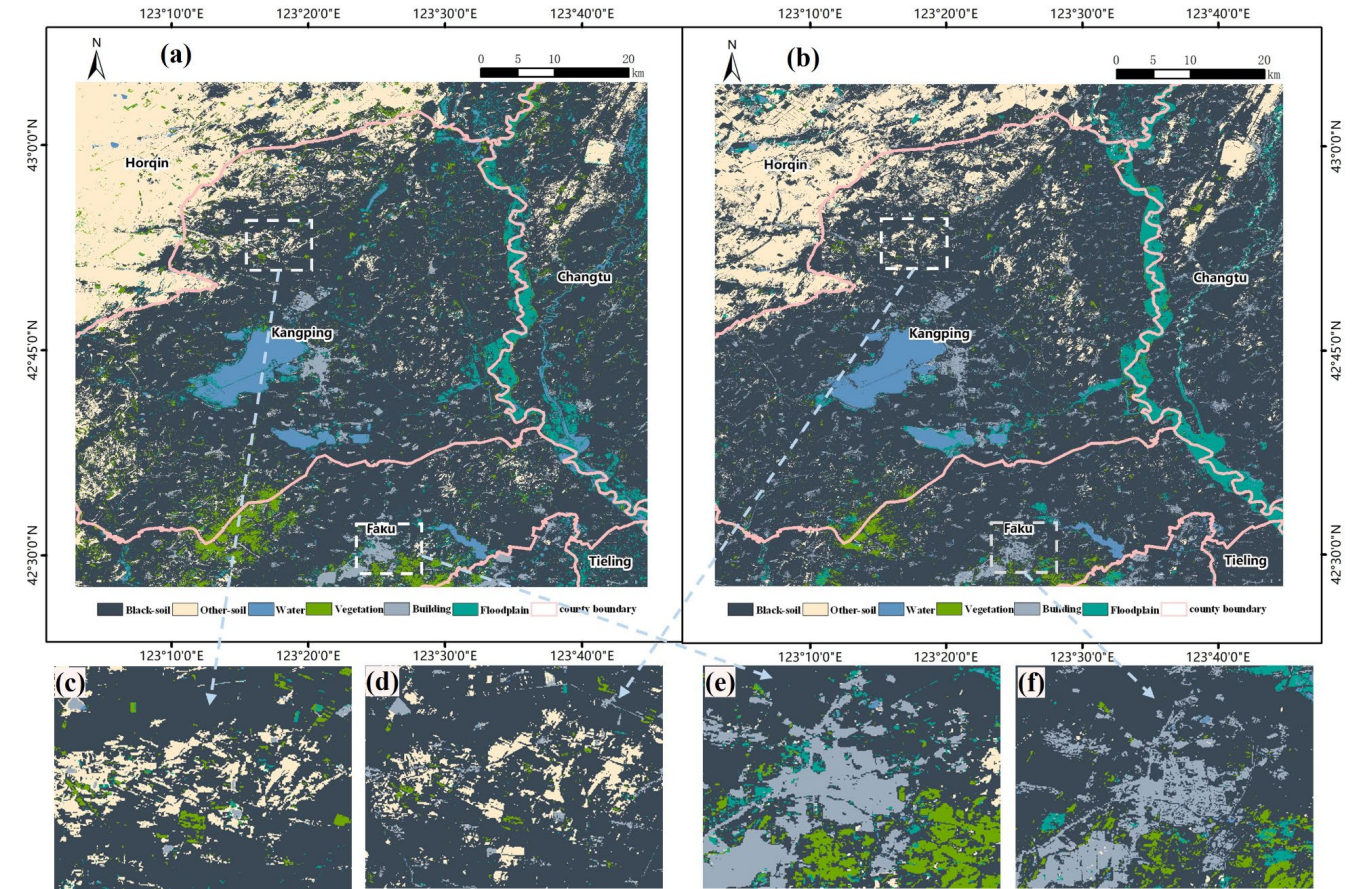
Model robustness evaluation (**a**) MEF-RF model result using Landsat-8 (**b**) MEF-RF model result using Sentinel-2 (**c**, **e**) MEF-RF model result using Landsat-8 in detail (**d**, **f**) MEF-RF model result using Sentinel-2 in detail, Figures were created using QGIS (version 3.20, https://www.qgis.org/).

According to the black soil distribution results in Fig. 10, the 30 m spatial resolution map of black soil distribution obtained from the Landsat-8 is prone to loss of detail information, compared to the 10 m resolution of the Sentinel-2. For example, buildings and vegetation in soil distribution areas are susceptible to under-scoring, and black soils in large-scale distribution areas of other soil types are easily misclassify for other soil types. However, the fact that the extent of black soil identified using different data sources is basically the same proved the robustness of the model developed in this study.

### Discussion

The multi-temporal ensemble features extracted in this paper could remove the invalid information from the image. The common features formed with the compression and dimensionality reduction of multiple remote image data could effectively reveal the surface information of black-soil resources. However, the consideration of alternative features in this study is still lacking. The spectral index should be constructed innovatively by selecting sensitive bands on the basis of studying the spectral characteristics of black soil resources. Furthermore, Since the accuracy of mapping black-soil is determined by the selection of classification features, it remains to be verified whether the classification features selected in this study can be applied successfully in other study areas.

The performance of model was different when using diverse geographic covariates. Although in this paper, the temperature covariates improved the model performance. The relationship between the covariates and the model in mapping black-soil resource is still a complex relationship to be further investigated. Finally, it is assumed in this study that high spatial resolution image has higher mapping accuracy. The application of the model on Landsat-8 obtained better performance and confirmed its robustness. However, there is still a lack of application cases of the model to higher spatial resolution satellites. These aspects still need attention and exploration.

## Conclusions

This study proposed a comprehensive and multispectral features-based approach built on a machine learning algorithm named multi-temporal ensemble features model (MEF-RF) for delineating the spatial extent of black-soil resources. The results showed that ensemble features from multi-temporal images can provide complementary information to improve the accuracy of black-soil discriminating. In addition, the effects of adding geographic covariates in MEF-RF model were evaluated in this study. It indicates that adding temperature covariate is another key means to improve model performance in mapping black-soil resource in the study area. Finally, we assume that the high spatial resolution image has a better effect of black soil mapping. Applied to slightly lower spatial resolution images, the MEF-RF model still has good performance. The results proved that the model improved in this study are generalizable to commonly used remote sensing images, which could be helpful for management of black-soil resources. Jianfang Hu conceived and designed the study and drafted the manuscript. Yulei Tang and Jianfang Hu carried out the experiments. All authors revised and edited the manuscript and approved the final version of the manuscript.

## Electronic supplementary material

Below is the link to the electronic supplementary material.

Supplementary Material 1

## Data Availability

All data relevant to the study are included in the article or uploaded as supplementary information. In addition, the datasets used or analyzed during the current study are available from the corresponding author on reasonable request.
