# Supplementary material for: Improving model performance in mapping black-soil resource with machine learning methods and multispectral features
Source: Sci Rep. 2025 Jan 7;15:1199. doi: 10.1038/s41598-024-82399-3 (PMC11706945; doi:10.1038/s41598-024-82399-3)
Supplement: Supplementary file 1 — Supplementary Material 1 [file 41598_2024_82399_MOESM1_ESM.pdf]

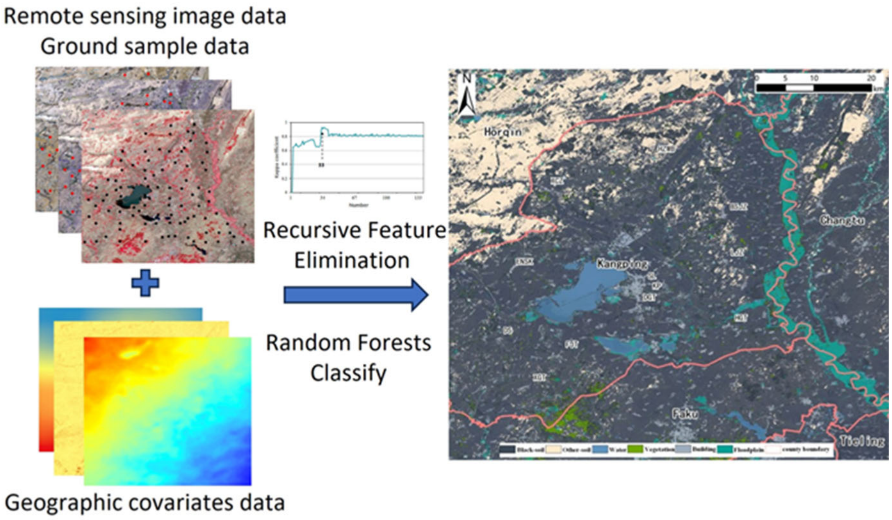

Supplementary Figure.1. Graphical abstract

Supplementary Table S1. Information of towns covered by the study area

name of town	abbreviation
Xiaochengzi Town	XCZ
Haizhouwobu Town	HZWB
Beisijiazi Town	BSJZ
Erniusuokou Town	ENSK
Kangping Town	KP
Liangjiazi Town	LJZ
Shengli Town	SL
Dongguantun Town	DGT
Haoguantun Town	HGT
Fangjiatun Town	FJT
Dongsheng Town	DS
Xiguan Town	XGT

Supplementary Table S2. Ground sample data (verification data left out are labeled with *)

FID	longitude	Latitude	Landcover code	Landcover type
1	123.36	42.62	0	Black-soil
2	123.63	42.58	0	Black-soil
3	123.56	42.55	0	Black-soil
4*	123.65	42.57	0	Black-soil
5*	123.65	42.58	0	Black-soil
6	123.42	42.57	0	Black-soil
7*	123.48	42.56	0	Black-soil
8	123.38	42.57	0	Black-soil
9*	123.31	42.55	0	Black-soil
10*	123.59	42.53	0	Black-soil
11*	123.55	42.55	0	Black-soil
12	123.59	42.54	0	Black-soil
13	123.70	42.55	0	Black-soil
14	123.66	42.57	0	Black-soil
15	123.40	42.57	0	Black-soil
16	123.44	42.96	0	Black-soil
17	123.50	43.02	0	Black-soil
18	123.43	42.94	0	Black-soil
19*	123.44	42.95	0	Black-soil
20	123.50	42.98	0	Black-soil
21	123.55	42.94	0	Black-soil
22	123.22	42.86	0	Black-soil
23*	123.34	42.84	0	Black-soil
24	123.40	42.91	0	Black-soil
25	123.45	42.91	0	Black-soil
26	123.56	42.91	0	Black-soil
27*	123.23	42.85	0	Black-soil

28	123.26	42.83	0	Black-soil
29	123.47	42.85	0	Black-soil
30	123.37	42.83	0	Black-soil
31*	123.25	42.84	0	Black-soil
32	123.30	42.83	0	Black-soil
33	123.55	42.84	0	Black-soil
34	123.29	42.81	0	Black-soil
35*	123.07	42.76	0	Black-soil
36*	123.32	42.77	0	Black-soil
37	123.52	42.76	0	Black-soil
38	123.11	42.76	0	Black-soil
39	123.21	42.76	0	Black-soil
40	123.25	42.76	0	Black-soil
41	123.41	42.77	0	Black-soil
42	123.51	42.82	0	Black-soil
43	123.56	42.77	0	Black-soil
44	123.21	42.73	0	Black-soil
45	123.31	42.73	0	Black-soil
46	123.40	42.72	0	Black-soil
47	123.07	42.70	0	Black-soil
48	123.47	42.71	0	Black-soil
49*	123.52	42.69	0	Black-soil
50	123.11	42.69	0	Black-soil
51*	123.26	42.68	0	Black-soil
52	123.31	42.69	0	Black-soil
53	123.40	42.69	0	Black-soil
54	123.45	42.69	0	Black-soil
55*	123.59	42.66	0	Black-soil
56	123.36	42.66	0	Black-soil

57	123.40	42.67	0	Black-soil
58*	123.48	42.64	0	Black-soil
59	123.55	42.64	0	Black-soil
60	123.10	42.61	0	Black-soil
61	123.21	42.62	0	Black-soil
62	123.30	42.62	0	Black-soil
63	123.41	42.62	0	Black-soil
64	123.45	42.62	0	Black-soil
65	123.06	42.55	0	Black-soil
66	123.11	42.55	0	Black-soil
67	123.21	42.55	0	Black-soil
68*	123.37	42.92	0	Black-soil
69	123.46	42.92	0	Black-soil
70	123.54	42.86	0	Black-soil
71	123.37	42.86	0	Black-soil
72	123.43	42.86	0	Black-soil
73	123.48	42.99	0	Black-soil
74	123.47	42.79	0	Black-soil
75	123.35	42.80	0	Black-soil
76*	123.49	42.95	0	Black-soil
77	123.15	42.57	0	Black-soil
78	123.18	42.78	0	Black-soil
79	123.21	42.75	0	Black-soil
80*	123.36	42.77	0	Black-soil
81	123.16	42.74	0	Black-soil
82	123.50	42.74	0	Black-soil
83	123.14	42.71	0	Black-soil
84*	123.28	42.67	0	Black-soil
85	123.47	42.63	0	Black-soil

86	123.08	42.67	0	Black-soil
87	123.17	42.67	0	Black-soil
88*	123.19	42.67	0	Black-soil
89	123.26	42.65	0	Black-soil
90	123.50	42.65	0	Black-soil
91*	123.32	42.72	0	Black-soil
92	123.21	42.60	0	Black-soil
93*	123.28	42.76	1	Water
94	123.30	42.76	1	Water
95*	123.31	42.75	1	Water
96*	123.26	42.74	1	Water
97	123.27	42.74	1	Water
98*	123.29	42.74	1	Water
99*	123.23	42.72	1	Water
100	123.23	42.69	1	Water
101	123.27	42.71	1	Water
102	123.25	42.77	1	Water
103	123.25	42.77	1	Water
104	123.31	42.64	1	Water
105*	123.31	42.65	1	Water
106	123.32	42.64	1	Water
107	123.40	42.64	1	Water
108	122.97	42.75	1	Water
109	122.96	42.74	1	Water
110*	122.96	42.73	1	water
111	122.93	42.64	1	Water
112	123.55	42.50	1	Water
113	123.54	42.51	1	Water
114	122.94	42.84	1	Water

115*	123.03	42.87	1	Water
116*	123.25	42.53	2	Vegetation
117	123.24	42.53	2	Vegetation
118	123.25	42.54	2	Vegetation
119	123.22	42.51	2	Vegetation
120	123.21	42.51	2	Vegetation
121	123.21	42.52	2	Vegetation
122	123.23	42.52	2	Vegetation
123*	123.23	42.52	2	Vegetation
124	123.23	42.52	2	Vegetation
125	123.29	42.57	2	Vegetation
126*	123.27	42.51	2	Vegetation
127	123.26	42.52	2	Vegetation
128	123.26	42.52	2	Vegetation
129*	123.25	42.5	2	Vegetation
130	123.20	42.46	2	Vegetation
131*	123.20	42.46	2	Vegetation
132	123.17	42.45	2	Vegetation
133	123.24	42.44	2	Vegetation
134	123.20	42.42	2	Vegetation
135	123.47	42.45	2	Vegetation
136*	123.48	42.46	2	Vegetation
137	123.50	42.46	2	Vegetation
138	123.49	42.46	2	Vegetation

139	123.47	42.40	2	Vegetation
140	123.47	42.40	2	Vegetation
141	123.51	42.45	2	Vegetation
142	123.47	42.45	2	Vegetation
143*	123.48	42.46	2	Vegetation
144	123.45	42.48	2	Vegetation
145	123.39	42.49	3	Building
146	123.39	42.48	3	Building
147*	123.39	42.48	3	Building
148	123.38	42.48	3	Building
149*	123.38	42.48	3	Building
150	123.37	42.46	3	Building
151*	123.37	42.46	3	Building
152*	123.36	42.45	3	Building
153	123.36	42.46	3	Building
154	123.35	42.46	3	Building
155	123.34	42.46	3	Building
156*	123.36	42.47	3	Building
157	123.36	42.47	3	Building
158*	123.35	42.47	3	Building
159	123.36	42.47	3	Building
160*	123.38	42.48	3	Building
161*	123.37	42.48	3	Building
162	123.42	42.50	3	Building
163	123.41	42.50	3	Building
164	123.42	42.51	3	Building
165	123.42	42.50	3	Building
166	123.43	42.50	3	Building

167*	123.42	42.51	3	Building
168	123.39	42.52	3	Building
169	123.39	42.52	3	Building
170*	123.33	42.73	3	Building
171	123.35	42.74	3	Building
172	123.35	42.74	3	Building
173	123.34	42.73	3	Building
174	123.33	42.82	3	Building
175*	123.33	42.81	3	Building
176	123.33	42.81	3	Building
177	123.32	42.81	3	Building
178	123.31	42.79	3	Building
179	123.32	42.8	3	Building
180	123.21	42.67	3	Building
181	123.21	42.84	3	Building
182	123.53	42.42	3	Building
183	123.54	42.42	3	Building
184	123.52	42.43	3	Building
185*	123.58	42.50	3	Building
186	123.57	42.50	3	Building
187	123.57	42.49	3	Building
188	123.55	42.44	3	Building
189*	123.54	42.45	3	Building
190	123.43	42.56	3	Building
191	123.43	42.56	3	Building
192*	123.09	42.97	4	Other-soil
193	123.11	42.97	4	Other-soil
194*	123.11	42.87	4	Other-soil
195	123.13	42.88	4	Other-soil

196	123.12	43.04	4	Other-soil
197*	123.14	43.04	4	Other-soil
198	123.15	43.04	4	Other-soil
199*	123.25	43.06	4	Other-soil
200	123.27	43.06	4	Other-soil
201	123.11	42.96	4	Other-soil
202	123.12	42.95	4	Other-soil
203	123.11	42.93	4	Other-soil
204*	123.12	42.94	4	Other-soil
205	123.14	42.93	4	Other-soil
206	123.15	42.97	4	Other-soil
207*	123.18	42.98	4	Other-soil
208	123.17	42.99	4	Other-soil
209	123.18	42.99	4	Other-soil
210	123.02	43.05	4	Other-soil
211*	123.07	43.05	4	Other-soil
212	123.01	42.95	4	Other-soil
213	123.03	42.95	4	Other-soil
214*	123.02	42.93	4	Other-soil
215	123.03	42.93	4	Other-soil
216	123.02	42.91	4	Other-soil
217*	123.06	42.89	4	Other-soil
218	123.07	42.89	4	Other-soil
219	123.09	42.91	4	Other-soil
220	123.10	42.91	4	Other-soil
221*	123.12	42.93	4	Other-soil
222	123.12	42.93	4	Other-soil
223	123.14	42.93	4	Other-soil
224*	123.15	42.93	4	Other-soil

225	123.17	42.94	4	Other-soil
226*	123.17	42.95	4	Other-soil
227	123.16	42.91	4	Other-soil
228*	123.18	42.91	4	Other-soil
229	123.12	42.86	4	Other-soil
230	123.14	42.87	4	Other-soil
231*	122.98	42.96	4	Other-soil
232	122.99	42.96	4	Other-soil
233	122.99	42.96	4	Other-soil
234	122.92	42.96	4	Other-soil
235*	122.92	42.95	4	Other-soil
236	122.92	42.95	4	Other-soil
237	122.92	42.95	4	Other-soil
238	123.11	42.88	4	Other-soil
239	123.12	42.86	4	Other-soil
240*	123.34	43.03	4	Other-soil
241	123.36	43.04	4	Other-soil
242	123.39	43.05	4	Other-soil
243	123.42	43.07	4	Other-soil
244*	123.44	43.05	4	Other-soil
245	123.68	42.99	4	Other-soil
246	123.70	43.00	4	Other-soil
247	123.63	42.90	4	Other-soil
248	123.64	42.90	4	Other-soil
249*	123.63	42.89	4	Other-soil
250*	123.69	42.98	4	Other-soil
251	123.74	43.07	4	Other-soil
252*	123.74	43.08	4	Other-soil
253	123.75	43.08	4	Other-soil

254	123.02	42.8	4	Other-soil
255*	123.02	42.81	4	Other-soil
256	122.95	42.87	4	Other-soil
257	122.93	42.96	4	Other-soil
258*	122.99	42.92	4	Other-soil
259	122.95	42.93	4	Other-soil
260	122.95	42.88	4	Other-soil
261*	123.04	42.81	4	Other-soil
262	123.66	42.63	5	Floodplain
263*	123.65	42.64	5	Floodplain
264	123.59	42.67	5	Floodplain
265*	123.59	42.67	5	Floodplain
266	123.58	42.77	5	Floodplain
267*	123.59	42.77	5	Floodplain
268	123.60	42.8	5	Floodplain
269	123.59	42.8	5	Floodplain
270*	123.59	42.81	5	Floodplain
271	123.59	42.84	5	Floodplain
272*	123.59	42.85	5	Floodplain
273	123.58	42.87	5	Floodplain
274*	123.58	42.89	5	Floodplain
275*	123.58	42.90	5	Floodplain
276	123.58	42.92	5	Floodplain
277*	123.56	42.95	5	Floodplain
278	123.56	42.98	5	Floodplain

279	123.56	43.00	5	Floodplain
280*	123.58	43.00	5	Floodplain
281	123.58	43.02	5	Floodplain
282*	123.60	43.06	5	Floodplain
283	123.56	42.96	5	Floodplain
284	123.59	42.94	5	Floodplain
285*	123.59	42.89	5	Floodplain
286	123.58	42.89	5	Floodplain
287	123.58	42.86	5	Floodplain
288*	123.58	42.80	5	Floodplain
289	123.78	42.48	5	Floodplain
290*	123.76	42.52	5	Floodplain
291	123.74	42.55	5	Floodplain
292*	123.67	42.60	5	Floodplain
293	123.35	42.73	5	Floodplain
294*	123.35	42.75	5	Floodplain
295	123.34	42.70	5	Floodplain
296*	123.34	42.70	5	Floodplain
297	123.38	42.74	5	Floodplain
298*	123.38	42.74	5	Floodplain

Supplementary Table S3. Band parameter information of Sentinel-2

Band	Description	Center Wavelength/nm	wavelength range /nm	Pixel Size/m
B1	Coastal	443.9	27	60
B2	Blue	496.6	98	10
B3	Green	560.0	45	10

B4	Red	664.5	38	10
B5	Red Edge	703.9	19	20
B6	Red Edge	740.2	18	20
B7	Red Edge	782.5	28	20
B8	NIR	835.1	145	20
B8A	Red Edge	864.8	33	20
B9	Water vapor	945.0	26	60
B10	SWIR-Cirrus	1373.5	75	60
B11	SWIR-1	1613.7	143	20
B12	SWIR-2	2202.4	242	20
QA60	Cloud mask			

Supplementary Table S4. Band parameter information of Landsat-8

Band	Description	Wavelength/nm	Pixel Size/m
SR_B1	ultra blue, coastal aerosol	435-451	30
SR_B2	Blue	452-512	30
SR_B3	Green	533-590	30
SR_B4	Red	636-673	30
SR_B5	NIR	851-879	30
SR_B6	SWIR-1	1566-1651	30
SR_B7	SWIR-2	2107-2294	30
SR_QA_AEROSAL	Aerosol attributes		

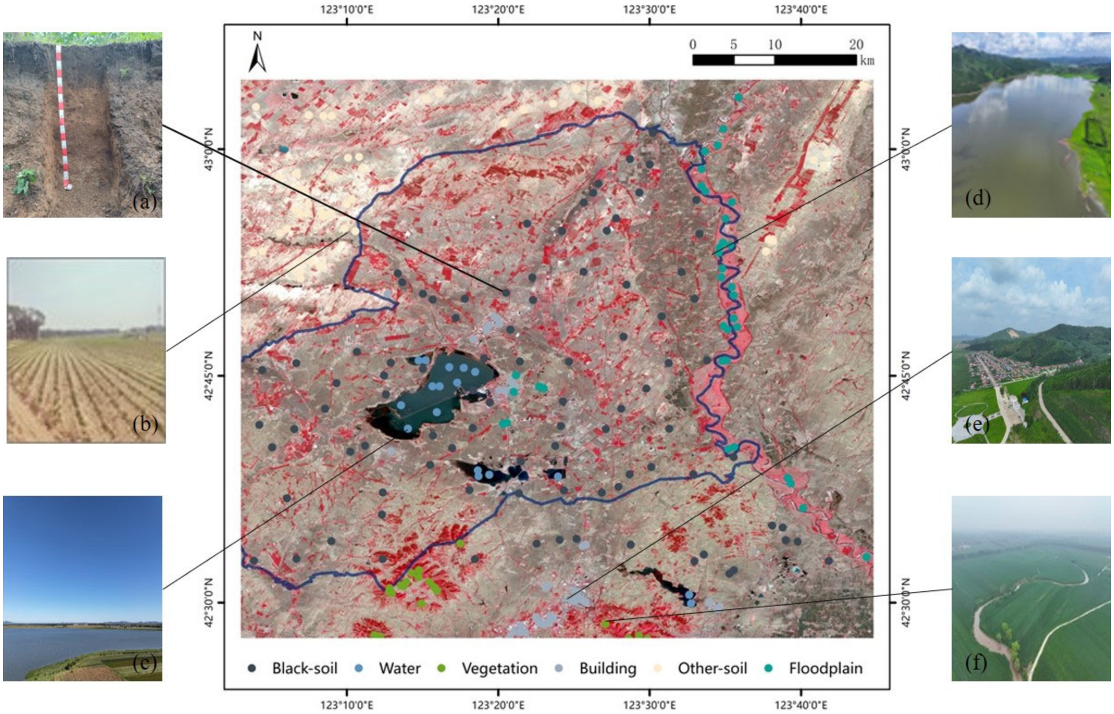

Supplementary Figure.2. Ground truth of different land use and land cover classes, (a) Black-soil, (b) Other-soil, (c) Water, (d) Vegetation, (e) Building, (f) Floodplain

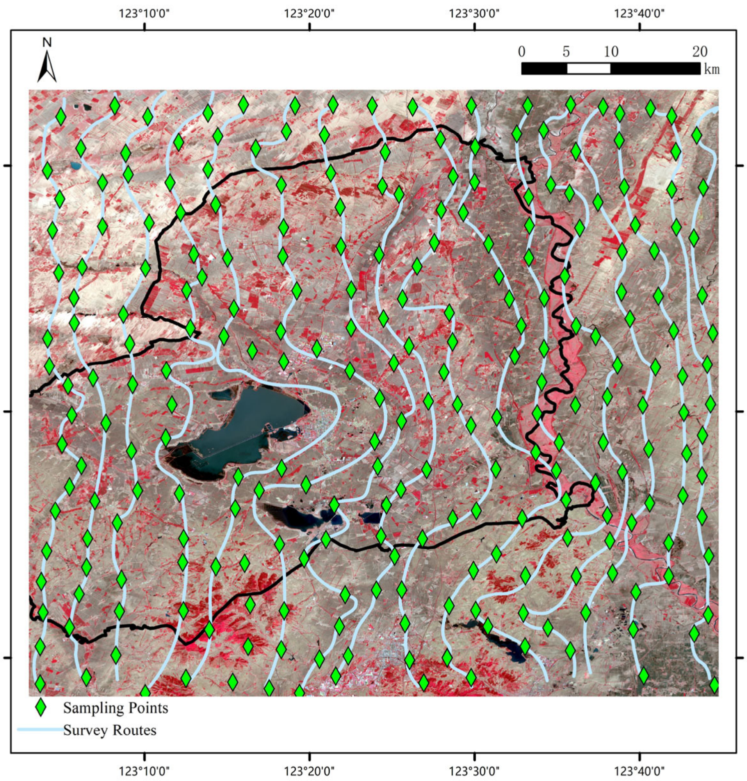

Supplementary Figure.3. Validation of the survey route

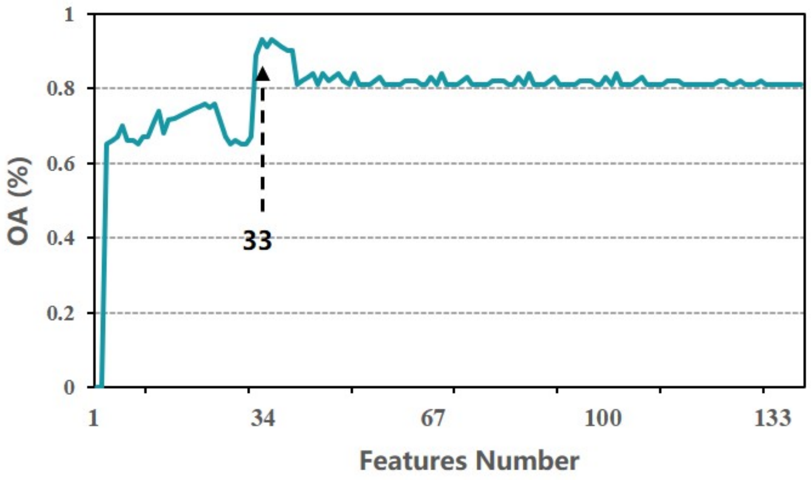

Supplementary Figure.4. Relationship between model performance and number of features
